# Protective Effect of *Citrus Medica limonum* Essential Oil against *Escherichia coli* K99-Induced Intestinal Barrier Injury in Mice

**DOI:** 10.3390/nu15122697

**Published:** 2023-06-09

**Authors:** Weixuan Tang, Zhuo Zhang, Dechao Nie, Yan Li, Shutian Liu, Yanling Li

**Affiliations:** Animal Science and Technology College, Beijing University of Agriculture, Beijing 102206, China; winsometang@163.com (W.T.); 202030321119@bua.edu.cn (Z.Z.); 202030312008@bua.edu.cn (D.N.); 202130321116@bua.edu.cn (Y.L.); 202130312009@bua.edu.cn (S.L.)

**Keywords:** *Citrus Medica limonum* essential oil, intestinal injury, mice, *Escherichia coli* K99

## Abstract

*Citrus Medica limonum* essential oil (LEO) has been reported to have antibacterial and anti-inflammatory activities, but its protective effect in the intestine remains unknown. In this study, we researched the protective effects of LEO in relation to intestinal inflammation induced by *E. coli* K99. The mice were pretreated with 300, 600, and 1200 mg/kg LEO and then stimulated with *E. coli* K99. The results showed that *E. coli* K99 caused immune organ responses, intestinal tissue injury, and inflammation. LEO pretreatment dose-dependently alleviated these changes by maintaining a low index in the thymus and spleen and producing a high content of immunoglobulin A, G, and M (IgA, IgG, and IgM) and low content of tumor necrosis factor-α (TNF-α), interleukin-1β (IL-1β), and interleukin-6 (IL-6). Intestinal integrity as a consequence of the LEO pretreatment may be related to the high mRNA expression of intestinal trefoil factor (ITF) and the low mRNA expression of transforming growth factor-β1 (TGF-β1). Conclusively, an LEO pretreatment can alleviate *E. coli* K99-induced diarrhea, immune organ response, and body inflammation in mice by reducing the levels of inflammatory cytokines and improving the levels of immunoglobulin, and the intestinal integrity remained highest when maintaining the high mRNA expression of ITF and keeping the mRNA expression of TGF-β1 low in the intestinal tissue.

## 1. Introduction

The health of calves is of great significance to beef cattle and dairy cattle farming. However, newborn calves are susceptible to gastrointestinal diseases due to the incomplete development of gastrointestinal immune barrier function, which affects growth performance and reduces production efficiency [1]. Among them, diarrhea is the most common disease in the breeding of newborn calves. The incidence of calf diarrhea in the lactation period is as high as 51.4%, and the mortality rate is as high as 72.8% [1]. Therefore, the prevention of diarrhea is an urgent problem to be solved in relation to calf production. The causes of calf diarrhea can be divided into two categories: biotic factors and abiotic factors. Abiotic factors include nutrition, management, and the environment, and biological factors include *Escherichia coli*, coronavirus, rotavirus, Cryptosporidium, etc. [2,3].

*Escherichia coli* (*E*. *coli*) is considered to be one of the most adaptable and pathogenic bacteria. It can cause a variety of infectious animal diseases, including gastrointestinal diseases and parenteral infections. At the same time, it is also part of the mammalian intestinal symbiotic flora [4]. The group of *Escherichia coli* that cause diarrhea is usually enteropathogenic *E. coli* (ETEC), among which *E. coli* K99 (*Escherichia coli* K99, 1975) is one of the most common bacteria that cause bacterial diarrhea in young ruminants [5,6]. *E. coli* K99 can induce inflammatory responses and diarrhea in calves by destroying the tight junction proteins of the mammalian intestinal epithelium and intestinal mucosal barrier [5,7,8].

Antibiotics are an effective means of treating/preventing diarrhea in calves. However, in recent years, environmental residues and drug-resistant pathogenic bacteria caused by the abuse of antibiotics have become a common problem in cattle production [9,10,11,12]. Therefore, many countries and regions have restricted the use of some antibiotics in animals and animal feed, and it is necessary to find new and safe alternatives to antibiotics. Plant extracts have attracted extensive attention from researchers due to their natural green origin and low resistance to drugs. Among them, plant essential oils have been widely studied because of their good antibacterial, anti-inflammatory, and biological safety [13].

There is a huge output of citrus plants in China (55.9561 million tons), and citrus essential oil (EO) is mainly extracted from citrus peel, which is a mixture of small molecules with various biological activities and is widely used in food, medicine, and other industries [14,15]. *Citrus Medica limonum* essential oil (LEO) is one of the EOs of citrus plants, and its main components are d-limonene, γ-terpinene, and β-pinene [16]. LEO has been reported to have antioxidant, antibacterial, anti-inflammatory, pain relief, and neuroprotective activities [14,17]. Studies have shown that LEO has good antibacterial properties against *E*. *coli* [18] and protective effects on the gastric mucosa and barrier function in mice [19,20]. In this study, we aim to further explore the protective effect of LEO on mammalian pathogenic *E*. *coli* K99-induced intestinal injury in mice and to provide a theoretical basis for the study of LEO in the treatment of bacterial diarrhea.

## 2. Materials and Methods

### 2.1. Ethic Approval

This study was conducted in accordance with the guidelines of the Beijing Laboratory Animal Management Regulations (2021 revision). All of the experimental procedures were approved by the Laboratory Animal Ethics Committee of Beijing University of Agriculture and conformed to the legal mandates and national guidelines regarding the care and maintenance of laboratory animals [SYXK 2021-0001, 2021.01.04].

### 2.2. Chemicals

LEO (purity 99%, extracted from *Citrus Medica limonum*, collected from Italy) was obtained from Nanjing VINCERO international trade Co., Ltd. (Nanjing, China). The RNA prep Pure Animal Tissue Total RNA Extraction Kit DP431, GoScript Reverse Transcription System A5000, and 2 × RealStar Green Power Mixture A311-01 were obtained from BOYOBIO LIFE SCIENCE Co., Ltd. (Beijing, China). The ITF and TGF-β1 primers were obtained from Sangon Biotechnology Co., Ltd. (Shanghai, China).

### 2.3. Chemical Composition of LEO

LEO (848 mg/mL) was diluted (1:50) in methanol and tested through the use of GC-MS QP2010 ultra (Shimadzu, Kyoto, Japan), as described previously [21]. The column DB-5MS (Agilent, J&W Scientific, 30 m × 0.25 mm × 0.25 μm, Santa Clara, CA, USA) inlet temperature was 250 °C, the gas split ratio was 10:1, the carrier gas was high-purity helium, and the flow rate was 1 mL/min. It was maintained at 40 °C for two minutes and then 10 °C/min to 300 °C for five minutes. The operating temperature of the ion source was 220 °C, and the operating temperature of the interface was kept at 280 °C. Within 3 min of the solvent removal time, m/z was scanned in a range of 45–500. Then, the sample was tested directly on the computer. The data were collected using MassHunter qualitative analysis software (Agilent Technologies, Santa Clara, CA, USA) with sample solvent used as a blank. The resulting data were screened and matched against NIST and other special standard chromatographic libraries to identify the individual chemical components in LEO. Each chemical component was subsequently quantified through the use of peak area normalization.

### 2.4. Animal Study

#### 2.4.1. LD_50_ of *E. coli* K99

Forty-eight Balb/C mice were fed (with water and feed ad libitum) for 3 days, and then all of the mice were randomly divided into 6 groups, with half male and half female. Each mouse in groups 1–5 was intraperitoneally injected with 1 mL of different concentrations of *E. coli* K99 solution (10^4^, 10^5^, 10^6^, 10^7^, and 10^8^ CFU/mL), and group 6 was injected with 1 mL of normal saline. Following this, 24 h after (d5) the challenge, the clinical symptoms of the experimental mice were observed, and the number of death was recorded. Finally, the LD_50_ values were calculated using a modified Coors method according to the following formula:

LD_50_ = log_−1_[Xm − i(ΣP − 0.5)] (Xm: Dose logarithm of the maximum dose group. i: Logarithm of the ratio of the high dose to the low dose for two adjacent groups (The difference between the logarithmic doses of two adjacent groups))(1)

#### 2.4.2. Animals and Experimental Treatment

Eighty-eight Balb/C mice (5 weeks, 20.0 ± 1.6 g, half male and half female) were obtained from Spefu (Beijing, China) Biotechnology Co., Ltd. (Beijing, China). The animals were housed under controlled temperature conditions (25 ± 1 °C, with water and feed ad libitum). Forty Balb/C mice (half male and half female, 6 weeks, 20.0 ± 1.6 g) were randomly divided into 5 groups as follows: the (1) CON group (normal saline + Tween 80), the (2) K99 group (injected *E. coli* K99), and the (3–5) EOL, M, and H groups (supplemented 300, 600, and 1200 mg/kg BW LEO, respectively, + *E. coli* K99). The dose of LEO was determined in our previous study [20], and LEO was dissolved in Tween 80 and subsequently diluted to 300, 600, and 1200 mg/kg with sterile distilled water. Before the test, all of the mice were fasted for 12 h, and then the mice in the CON group and K99 group were given the same amount of normal saline, and the LEO group was given three different doses of LEO via intragastric gavage for 7 consecutive days. On the 7th day, 1 h after gavage, mice in the CON group were intraperitoneally injected with normal saline, and the mice in the K99 and EO groups were intraperitoneally injected with a concentration corresponding to 10^8^ CFU/mL of *E. coli* K99. The diarrhea rates of the mice in each group were observed and recorded within 6 h. After 6 h, the blood samples of all groups were collected from the eyeballs and then centrifuged to obtain the serum and frozen at 4 °C for the analysis of immunoglobulin A, M, and G (IgA, IgM, and IgG) levels and tumor necrosis factor-α (TNF-α), interleukin-6 (IL-6), and interleukin-1β (IL-1β) levels. The spleen, thymus, and duodenum of each mouse were dissected, the spleen and thymus were weighed, and the duodenum was divided into two parts; one part was placed in 10% formalin solution for histopathological analysis, and the other part was frozen at −80 °C for the analysis of the mRNA expression of intestinal trefoil factor (ITF) and transforming growth factor-β1 (TGF-β1).

#### 2.4.3. Immune Organs Index in Mice

The spleen and thymus were weighed using an analytical balance (Sartorius BSA124S, Gottingen, Germany). The effect of *E. coli* K99 and LEO on immune organs were calculated according to the formula, as described by Xu et al. [22]:

Spleen/Thymus index = spleen/thymus weight (mg)/body weight (g)(2)

#### 2.4.4. Histopathology

The effects of *E. coli* K99 and LEO on the gut (duodenum) were evaluated using hematoxylin–eosin (H&E, Sigma, St. Louis, MO, USA). The anterior segment of the duodenum tissue was washed with normal saline and fixed in 4% paraformaldehyde, and treated through a series of ethanol concentrations and embedded in paraffin. Subsequently, the duodenum tissue slice (5 mm) was stained with hematoxylin and eosin (HE) and evaluated under an inverted fluorescent microscope WYS-41XDY (VIYEE, VIYEE photoelectric devices Co., Ltd., Tianjin, China).

#### 2.4.5. Blood Immune Indexes and Inflammatory Factors in Mice

The serum immune index (IgA, IgM and IgG levels) and inflammatory factors (TNF-α, IL-1β, and IL-6) were determined using an enzyme-linked immunosorbent assay and through the use of an ELISA Kit (Beijing Sino-UK Institute of Biological Technology, Beijing, China).

#### 2.4.6. mRNA Relative Expression of ITF and TGF-β1 in Duodenum

The posterior segment of the duodenum tissue was isolated for the evaluation of the mRNA expression of ITF and TGF-β1. The duodenum tissue was thoroughly homogenized, and the total RNA was extracted according to the RNA Prep Pure animal tissue total RNA extraction kit DP431 (Tiangen Biochemical, Beijing, China). The concentration and purity of the extracted RNA were determined using an ultra-microspectrophotometer DS-11 (DeNovix, Wilmington, CA, USA) and ultraviolet spectrophotometer UV2400 (Sunny Hengping Instrument, Shanghai, China), respectively, and then the cDNA was synthesized according to the GoScript™ Reverse Transcription System Reverse Transcription Kit A5000 (Promega, Beijing, China). Finally, real-time PCR was performed using 2×RealStar Green Power Mixture A311-01 (Genstar, Beijing, China). The relative mRNA expression levels were calculated using the 2^−ΔΔCt^ method. The PCR primer sequences are shown in Table 1.

### 2.5. Statistical Analysis

GraphPad Prism 9.4.1 (GraphPad Software FI., Boston, MA, USA) and Origin 2019 (OriginLab, Northampton, MA, USA) were used for the statistical analysis of the data and drawing the graph of the LD_50_ values of *E. coli* K99, the mouse diarrhea rate, organ index, and the relative mRNA expressions of the related cytokines. Eight mice from each treatment group were selected for triplicate data analysis per mouse, and the results are expressed as mean + SD. SAS 9.4 (SAS Inst. Inc., Cary, NC, USA), which was used to analyze the data. A Shapiro–Wilk w test was used to analyze the normality of the data, the significance of the differences between the two experimental groups was assessed using a one-way ANOVA, and the dose effect of LEO was tested via linear and quadratic orthogonal comparisons, and probit regression models were used to calculate the LD_50_ values of *E. coli* K99. *p* ≤ 0.05 was considered significant, and *p* ≤ 0.01 was considered highly significant.

## 3. Results

### 3.1. Chemical Composition of LEO

GC-MS showed that the LEO contained about 50 components (Table 2). The dominant component was D-limonene (47.19%); the other main components included β-pinene (12.79%) and γ-terpinene (11.49%), while the other remaining components were less part of the total LEO (23.58%).

### 3.2. LD_50_ of E. coli K99 and Diarrhea Prevention Effect of LEO

**Figure 1:**
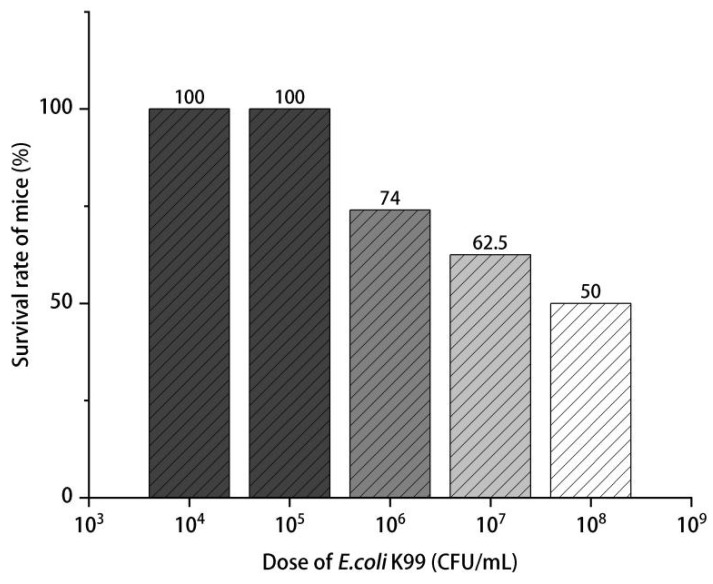
*E. coli* K99 concentrations and mice mortality (*n* = 8).

**Figure 2:**
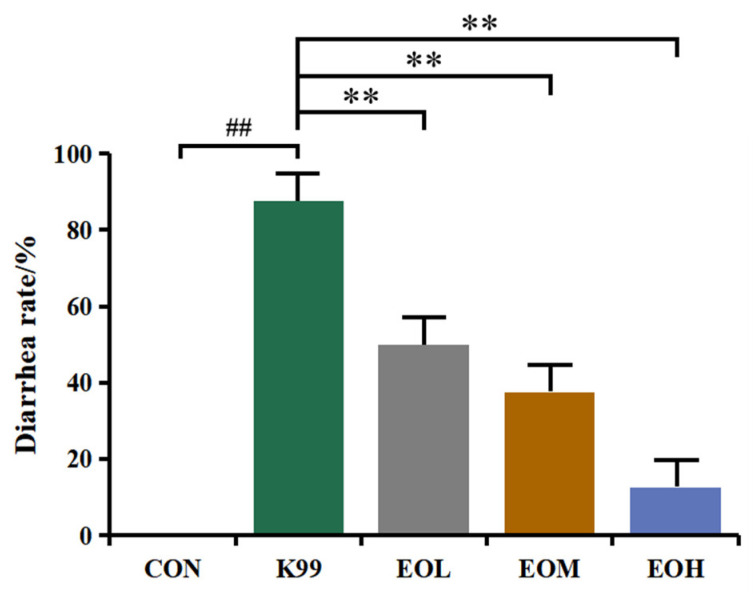
Diarrhea rate of mice under different treatments. The diarrhea rate was calculated by using the ratio of the number of mice with diarrhea to the total number of mice in each group (*n* = 8). Control: treated with normal saline; K99: challenged with 10^8^ CFU/mL *E. coli* K99; EOL: treated with 300 mg/kg *C. Medica limonum essential oil* + *E. coli* K99; EOM: treated with 600 mg/kg *C. Medica limonum* essential oil + *E. coli* K99; EOH: treated with 1200 mg/kg *C. Medica limonum* essential oil + *E. coli* K99. ##, *p* < 0.01, means the *E. coli* K99 group was significantly different from the Control group; **, *p* < 0.01, means that the significant difference between different doses of *C. Medica limonum* essential oil group and the *E. coli* K99 group.

The LD_50_ values were measured to confirm the medium lethal concentration. During the experimental period, the *E. coli* K99 treatment resulted in different degrees of diarrhea and death of mice in each treatment group, and the concentration of *E. coli* K99 and the corresponding mortality of mice are shown in Figure 1. The mice treated with 10^6^, 10^7^, and 10^8^ CFU/mL *E. coli* K99 had the most serious diarrhea, which was accompanied by refusing food and slow movement; the diarrhea degree of mice in the 10^4^ and 10^5^ CFU/mL *E. coli* K99 groups was less severe. Moreover, there was a dose-dependent relationship between *E. coli* K99 concentration and mouse mortality, with an LD_50_ of 10^8^ CFU/mL established for subsequent experiments. The diarrhea prevention results of LEO are shown in Figure 2. The diarrhea rate of mice in the *E. coli* K99 group was 87.5%, whereas the EOL, EOM, and EOH groups were 50%, 37.5%, and 12.5%, respectively. The diarrhea rate decreased linearly with increasing LEO dose.

### 3.3. Effects of LEO and E. coli K99 on Organ Indexes in Mice

**Figure 3:**
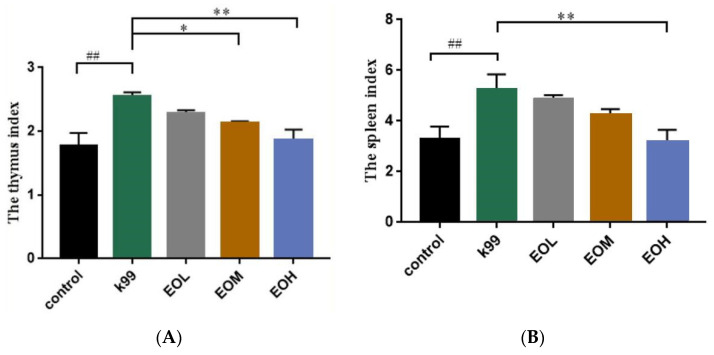
Effect of *C. Medica limonum* essential oil on immune organ index of *E. coli* K99-induced immune response in mice (*n* = 8/group). (**A**) Thymus index; (**B**) Spleen index. Control: treated with normal saline; K99: challenged with *E. coli* K99; EOL: treated with 300 mg/kg *C. Medica limonum* essential oil + *E. coli* K99; EOM: treated with 600 mg/kg *C. Medica limonum* essential oil + *E. coli* K99; EOH: treated with 1200 mg/kg *C. Medica limonum* essential oil + *E. coli* K99. Data were presented as mean ± standard deviation (SD). ##, *p* < 0.01, a significant difference between *E. coli* K99 group and control group; *, *p* < 0.05 and **, *p* < 0.01, a significant difference between *C. Medica limonum* essential oil group and *E. coli* K99 group.

The spleen and thymus index were calculated to evaluate the overall impaction of LEO and *E. coli* K99 on the immune status of mice, and the relative results are shown in Figure 3. Compared with the control group, the mice in the *E. coli* K99 group had higher spleen and thymus indexes (*p* < 0.01). Mice under the 600 mg/kg LEO pretreatment had a lower thymus index (*p* < 0.05), and mice in the 1200 mg/kg LEO group had lower thymus and spleen indexes (*p* < 0.01). The spleen and thymus indexes of the infected mice decreased linearly with the increase in LEO concentration (*P_L_* < 0.01).

### 3.4. Effect of LEO and E. coli K99 on the Morphology of Duodenal Tissue in Mice

**Figure 4:**
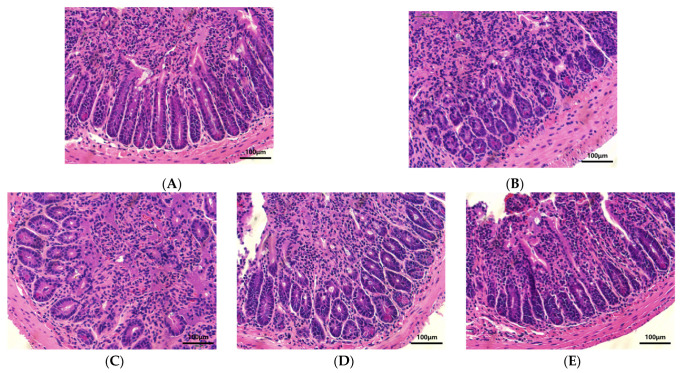
Histology of duodenal and intestinal villi of experiment mice (*n* = 8/group). Structure and profile changes of duodenal and intestinal villi with different treatments: (**A**) treated with normal saline, (**B**) challenged with *E. coli* K99, (**C**) treated with 300 mg/kg *C. Medica limonum* essential oil + *E. coli* K99, (**D**) treated with 600 mg/kg *C. Medica limonum* essential oil + *E. coli* K99, and (**E**) treated with 1200 mg/kg *C. Medica limonum* essential oil + *E. coli* K99 (scale bar = 100 µm).

For the evaluation of the changes in the intestinal tissue structure after *E. coli* K99 and LEO treatment in mice, the pathological section of the duodenal and intestinal villi was observed, and the result is shown in Figure 4. The morphology of the duodenal and intestinal villi was neatly arranged, slender, and tight, and the profile of the duodenal and intestinal villi was complete and clear in the mice of the control group (Figure 4A). The *E. coli* K99 challenge destroyed the integrity and tight junction of the duodenal and resulted in the breakage of the intestinal villi (Figure 4B). The duodenal and intestinal villi structure of the infected mice was relatively complete under the pretreatment of 600 and 1200 mg/kg LEO, while 300 mg/kg LEO administration performed a weak effect in protecting the structure injury of duodenal and intestinal villi (Figure 4C). Furthermore, 300 mg/kg of LEO showed the same effect in terms of immune function and inflammatory results upon histopathology.

### 3.5. Effects of LEO and E. coli K99 on Immune and Inflammatory Factors in Blood

**Figure 5:**
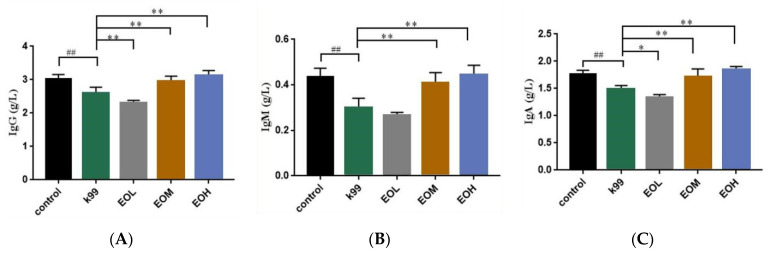
Effects of *C. Medica limonum* essential oil and *E. coli* K99 on immune indexes in mice (*n* = 8/group). (**A**) IgG, (**B**) IgM, and (**C**) IgA levels in serum. Control: treated with normal saline; K99: challenged with *E. coli* K99; EOL: treated with 300 mg/kg *C. Medica limonum* essential oil + *E. coli* K99; EOM: treated with 600 mg/kg *C. Medica limonum* essential oil + *E. coli* K99; EOH: treated with 1200 mg/kg *C. Medica limonum* essential oil + *E. coli* K99. Data were presented as mean ± SD. ##, *p* < 0.01, means the *E. coli* K99 group was significantly different from the Control group; *, *p* < 0.05 and **, *p* < 0.01, means that the significant difference between different doses of *C. Medica limonum* essential oil group and the *E. coli* K99 group.

To evaluate the effect of LEO and *E. coli* K99 on the blood immune index in mice, serum IgG, IgM, and IgA levels were measured, and the results are shown in Figure 5. Compared with the control group, the immunoglobulin levels of mice were significantly decreased after the *E. coli K99* challenge (*p* < 0.01). The 600 and 1200 mg/kg LEO pretreatment significantly improved the immunoglobulin levels of mice induced by *E. coli* K99 (*p* < 0.01), while 300 mg/kg LEO administration resulted in lower IgA (*p* < 0.05) and IgG (*p* < 0.01) levels than the *E. coli* K99 treatment. Furthermore, the levels of IgA, IgM, and IgG in the serum of infected mice exhibited a linear increase with escalating doses of LEO (*P_L_* < 0.01).

**Figure 6:**
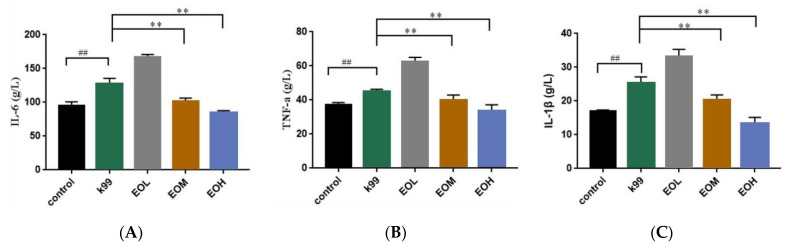
Effects of *C. Medica limonum* essential oil on *E. coli* K99-induced inflammation in mice (*n* = 8/group). (**A**) IL-6, (**B**) TNF-α, and (**C**) IL-1β levels in the serum of mice. Control: treated with normal saline; K99: challenged with *E. coli* K99; EOL: treated with 300 mg/kg *C. Medica limonum* essential oil + *E. coli* K99; EOM: treated with 600 mg/kg *C. Medica limonum* essential oil + *E. coli* K99; EOH: treated with 1200 mg/kg *C. Medica limonum* essential oil + *E. coli* K99. Data were presented as mean ± SD. ##, *p* < 0.01, means the *E. coli* K99 group was significantly different from the Control group; **, *p* < 0.01, means the significant difference between different doses of *C. Medica limonum* essential oil and *E. coli* K99 group.

The blood inflammatory cytokines levels were measured to evaluate the effect of LEO on *E. coli* K99-induced mice, and the results are shown in Figure 6. Compared with the control group, the *E. coli* K99 challenge significantly increased (*p* < 0.01) the TNF-α, IL-1β, and IL-6 levels in the serum. Under the pretreatment of 600 and 1200 mg/kg LEO, the TNF-α, IL-1β, and IL-6 levels in the serum of the infected mice were significantly decreased (*p* < 0.01), while 300 mg/kg LEO administration failed to resist the altered TNF-α, IL-1β, and IL-6 levels in the serum. Moreover, the LEO pretreatment dose-dependently decreased the IL-6, IL-1β, and TNF-α content in the serum of infected mice (*P_L_* < 0.01).

### 3.6. Effect of LEO and E. coli K99 on mRNA Relative Expression of ITF and TGF-β1 in Duodenum of Mice

**Figure 7:**
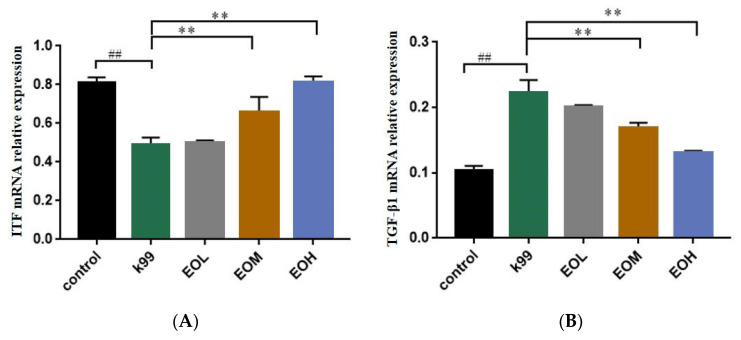
Effects of *E. coli* K99 and *C. Medica limonum* essential oil on mRNA relative expression levels of ITF and TGF-β1 in the duodenum of mice (*n* = 8/group). (**A**) ITF mRNA relative expression. *P_Q_* < 0.01, a quadratic increase as the concentration of *C. Medica limonum* essential oil increases. (**B**) TGF-β1 mRNA relative expression, *P_Q_* < 0.01, a quadratic decreasing curve as the concentration of *C. Medica limonum* essential oil increases. Control: treated with normal saline; K99: challenged with *E. coli* K99; EOL: treated with 300 mg/kg *C. Medica limonum* essential oil + *E. coli* K99; EOM: treated with 600 mg/kg *C. Medica limonum* essential oil + *E. coli* K99; EOH: treated with 1200 mg/kg *C. Medica limonum* essential oil + *E. coli* K99. Data were presented as mean ± SD. ##, *p* < 0.01, compared with the control group; **, *p* < 0.01, compared with the *E. coli* k99 group.

To explore the protective mechanism of LEO on intestinal tissue integrity, the effect of ITF and TGF-β1 relative expression on the mRNA was measured, and the results are shown in Figure 7. Compared with the control group, the mRNA relative expression of ITF was significantly decreased (*p* < 0.01), while TGF-β1 significantly increased (*p* < 0.01) after the *E. coli* K99 challenge in the intestinal tissue. Furthermore, 600 and 1200 mg/kg LEO showed an excellent protective effect on the normal mRNA expression of ITF and TGF-β1 in the intestinal tissue, while the 300 mg/kg LEO pretreatment failed to maintain the mRNA expression of ITF and TGF-β1 in intestinal tissue. Additionally, LEO dose-dependently increased ITF mRNA expression (*p* < 0.01) and decreased TGF-β1 mRNA expression (*p* < 0.01).

## 4. Discussion

ETEC can cause inflammatory diarrhea in young mammals, and its mechanism is that *E. coli* releases enterotoxins to destroy the tight junction of the host’s intestinal epithelium, damaging intestinal epithelial cells and inducing inflammatory responses in the host’s body [4,7]. According to the relative study, the diarrhea rate of the mice treated with 2 × 10^7^ CFU/mL *E. coli* ATCC25922 was 50% within 6 days [23]. In our study, compared with the control group, the diarrhea rate of *E. coli* K99-treated mice was significantly increased, and the LEO treatment dose-dependently reduced the diarrhea rate of *E. coli* K99-stimulated mice. The mortality rate of mice was 50%, and the diarrhea rate was 87.5% within 2 days after the 10^8^ CFU/mL *E. coli* K99 challenge, which means that *E. coli* K99 has pathogenicity in mice intestines. Based on the above results, 10^8^ CFU/mL *E. coli K99* conformed to the computational description of the LD_50_ values and can be used as a stimulation concentration for subsequent tests.

The thymus and spleen are important immune organs in mammals, and a variety of immune cells (macrophages and T lymphocytes) contained in them play important roles in the body’s innate immunity [24,25]. The initial assessment of organ immunity and intestinal morphological changes in mice can be visualized through the use of spleen and thymus indexes and intestinal histopathological sections. In this study, *E. coli* K99 infection caused immune responses in the spleen and thymus and histopathological damage to the intestinal tissue. With the gavage of low, medium, and high concentrations of LEO, the above changes in terms of immune organs and intestinal tissue were alleviated, which showed that pretreatment with LEO could protect the immune organs and intestinal tissue of mice challenged by *E. coli* K99.

Immunoglobulin is a kind of heterodimeric protein secreted by B-cell, among which immunoglobulin A, G, and M play important roles in forming the body’s primary immunity and protecting the mucosa from toxins and pathogenic microorganisms [25,26]. In our studies, the mice developed severe diarrhea, and the contents of IgA, IgG, and IgM in the serum were decreased after stimulation with *E. coli* K99, indicating that *E. coli* K99 successfully caused immune responses in mice. Mice that underwent an LEO pretreatment had higher IgA, IgM, and IgG contents in the serum, and the contents of IgA, IgM, and IgG increased with increasing LEO concentration simultaneously. It has been reported that citrus EOs have a good effect on improving the immune performance of infected mice [27,28], which may be derived from the effect of citrus EOs on enhancing the secretory activity of immune cells through immune stimulation [29]. D-limonene has been reported to regulate cellular lipid metabolism and immune function by changing the expression of the protein disulfide isomerase family, a member three pseudogene, and altering the expression of deacetylated histones [30]. The antibacterial activity of citrus EOs performed via reducing the production of enterotoxins by inhibiting the amount of *E. coli* K99, IgA, IgG, and IgM are mainly involved in the body’s defense against the invasion of pathogenic microorganisms and the neutralization of cytotoxins [26]. The increase in serum immunoglobulins may be related to the decrease in enterotoxin and the increase in the viability of mouse immune cells.

TNF-α is a proinflammatory factor that plays an important role in mammalian immunity and cell homeostasis. Its excessive secretion can cause intestinal inflammation and induce intestinal barrier damage [31,32]. Interleukin-6 (IL-6) is secreted by a variety of cells, and it can play an anti-inflammatory function by binding to the IL-6 receptor on the cell membrane; on the other hand, it can also bind to soluble IL-6 and subsequently recruit membrane-bound glycoproteins to promote inflammation [33]. Interleukin-1β (IL-1β) is a multifunctional proinflammatory cytokine that can promote the increase in permeability of intestinal epithelial cells in intestinal inflammation, leading to damage to the intestinal barrier [34]. Our results showed that the levels of TNF-α, IL-1β, and IL-6 in the serum increased significantly after stimulation with *E. coli* K99, indicating that *E. coli* K99 successfully caused inflammatory responses in mice. Mice treated with LEO showed a linear reduction in serum levels of TNF-α, IL-6, and IL-1β, which showed the alleviating effect of LEO with dose-dependent in terms of inflammation. Some EOs have the ability to alleviate intestinal inflammation, such as cinnamaldehyde, which can alleviate intestinal inflammation in mice and reduce the expression of TNF-α and IL-6 genes in intestinal tissue [35]; *Zanthoxylum bungeanum* EO could alleviate intestinal inflammation caused by *E. coli* by reducing the expression of TNF-α and IL-8 in intestinal tissues [23], indicating that EOs and their main components have a function in regulating inflammatory factors and anti-inflammatory activities. Regarding citrus EOs, Shen et al. [36] have reported that 250 μg/mL citrus EOs can reduce the IL-1β, TNF-α, and IL-6 secretion of RAW264.7 cells induced by LPS. Kummer et al. [37] have reported that 500 mg/kg D-limonene reduced the TNF-α levels in the peritoneal exudate of mice. Zhao et al. [20] have reported that 600 mg/kg LEO can alleviate the intestinal inflammation and injury induced by *E. coli* ATCC25922. However, in this study, 300 mg/kg LEO showed enhanced effects on the production of three inflammatory cytokines, which we speculate may be related to the active ingredient and the action dose of citrus EOs. Some citrus EOs do not have anti-inflammatory effects at low concentrations [38], and Zhao et al. reported that D-limonene, the main active component of LEO, also had a slight promoting effect in terms of *E. coli*-induced intestinal inflammation in mice at a dosage of 300 mg/kg [20]. Based on the above results, citrus EOs have an effect in alleviating intestinal inflammation caused by ETEC, which may derive from the dosage of LEO and the excellent anti-inflammatory effect of the chemical components, such as D-limonene and pinene in LEO [36,37].

Studies have reported that *E. coli* K99 can destroy intestinal physiological function [7,39]. Intestinal trefoil factor (ITF) is an important factor regulating intestinal epithelial recovery, which can be secreted by specific secretory cells in the intestine to the mucosal surface in large amounts and is believed to promote intestinal epithelial cell migration [40]. Transforming growth factor-β1 (TGF-β1) is a cytokine produced by a variety of inflammatory cells and non-inflammatory cells. It can act on almost all intestinal mucosal cells by activating intracellular Smad2/3 protein and inhibiting immune responses, which has a dual effect of promoting/inhibiting cellular inflammation [41]. In our results, the pathological damage of intestinal tissue might be caused by the abnormal expression of ITF and TGF-β1, which leads to the impaired repair function of the intestinal epithelium and the inflammation of the intestinal tissue. With increased levels of LEO pretreatment concentration, the microscopic structure of the duodenum of mice was significantly improved, the mRNA expression of ITF in intestinal tissue linearly increased, and the mRNA expression of TGF-β1 linearly decreased, showing that the LEO pretreatment maintained intestine integrity via promoting intestinal epithelial recovery and reducing TGF-β1-mediated inflammation. The regulatory effect of EOs on TGF-β1 in mouse intestinal tissues may be derived from the main chemical components, such as the fact that thymol can alleviate the intestinal mucositis by inhibiting the 5-FU-induced expression of NF-κB, TNF-α, and TGF-β1 in rats [42]. D-limonene has also been reported to inhibit TNF-α and TGF-β1 mRNA expression and alleviate hepatitis in rats [43]. According to related research, EOs from patchouli and tangerine peel mixed at a 1:2 ratio can increase the expressions of epidermal growth factor (EGF) and trefoil factor 2 (TFF2) protein in gastric tissues and improve gastric mucosal injury [44]; even in EO mixtures, citrus EOs (tangerine peel) were a substantial component with gastrointestinal protective activity, suggesting that citrus EOs have a good gastrointestinal trefoil factor regulation effect. TGF-β1 can protect intestinal epithelial IgA immune function [45] and down-regulate intestinal epithelial IL-6 signaling [46] through the receptor 1/SMAD2/3 pathway, indicating that the LEO dose-dependent increase in serum immunoglobulin content in mice may be related to the linear decrease in TGF-β1 expression because the high concentration of LEO protects the mouse intestine from *E. coli* K99 infection, and the intestinal epithelial demand for TGF-β1 regulatory immune function may be less than that of the *E. coli* K99 stimulation group and the low concentration in the EOs group. In addition, our previous study reported that 600 mg/kg LEO can protect the intestinal epithelial barrier of mice via increasing ZO-1, claudin, and occludin mRNA expressions [20]. TGF-β1 has the effect of down-regulating TNF-α levels, alleviating ZO-1 and occludin protein changes induced by TNF-α and protecting the intestinal epithelial barrier [47], indicating that LEO pretreatment may maintain the normal low expression of TGF-β1 in the intestinal tissue by maintaining the expression of intestinal-barrier-related proteins and reducing the expression of TNF-α in intestinal tissue. Otherwise, LEO dose-dependently increased ITF expressions in intestinal tissues can be attributed to the antibacterial activity of EOs on *E. coli* K99, and ITF is mainly involved in maintaining the integrity of the intestinal barrier by restricting intestinal tight junction proteins [48], suggesting that the high expression of ITF in intestinal tissues in this study is a normal manifestation of intestinal tissue.

Therefore, we speculate that the integrity of intestinal tissues after LEO preconditioning may be related to the high expression of ITF and the low expression of TGF-β1 in intestinal epithelial cells. However, the specific pathway through which LEO regulates the expression of ITF and TGF-β1 is still unclear. The current reference studies show that ITF and TGF-β1 are likely to be related to the activation of intestinal NF-κB. Whether LEO can protect intestinal tissue through other pathways has not been further investigated in this study. Meanwhile, the antibacterial activity of LEO can reduce *E. coli* K99 in the intestine and its adverse stimulation of intestinal tissue and plays an auxiliary anti-inflammatory and anti-injury role. In this study, we chose *E. coli* K99, which is one of the most common pathogens that causes diarrhea in newborn calves and is also more representative and pathogenic than *E. coli* ATCC 25922, to explore the efficacy of LEO in terms of preventing diarrhea in calves [5]. We found the protective function of LEO to *E. coli* K99 induced intestinal barrier damage from the perspective of the body’s immune system, inflammation, and intestinal barrier relative cytokine. To explain the protective effect of LEO against *E. coli* K99 preliminarily, we chose mice as an alternative to calves and revealed the degree of intestinal injury induced by *E. coli* K99 and the protective effect of LEO so as to have a more scientific basis for guiding calf research, and our results have shown that LEO has good anti-inflammatory activity and immune-adjusting function; furthermore, it has a protective capacity on intestinal injury induced by *E. coli* K99. Our results are considered to provide a partial theoretical basis on the use of LEO as an alternative to antibiotics for the treatment of calf diarrhea.

## 5. Conclusions

In conclusion, our study showed that *E. coli* K99 infection caused diarrhea, destroyed the structure of intestinal tissue, and induced immune injury and inflammation in mice. LEO has excellent anti-inflammatory activity in terms of protecting mouse intestinal tissue from *E. coli* K99 invasion, which works by protecting immune organs and intestinal tissue, maintaining a high content of IgA, IgG, and IgM and a low content of IL-6, IL-1β, and TNF-α in the serum. In addition, LEO pretreatment may protect intestinal integrity by maintaining the low mRNA expression of TGF-β1- and the high mRNA expression of ITF. Our research provides a theoretical basis for the application of LEO to protect calves from the diarrhea caused by *E. coli* K99.

## Figures and Tables

**Table 1:** Primer sequences used in RT-PCR.

Gene	Primer Sequence (5′-3′)
β-actin	F: CTGGAACGGTGAAGGTGACA R: AAGGGACTTCCTGTAACAATGCA
ITF	F: CTGTGCAGTGGTCCTGAAGC R: TTGGAGACAGGCCAACGTAA
TGF-β1	F: CCCCTGCAAGACCATCGAC R: CTGGCGAGCCTTAGTTTGGAC

**Table 2:** Main chemical components and relative contents of *C. Medica limonum* essential oil.

Peak Pegasus	Retention Time RI/min	Chemical Compound	Relative Amount/%
1	6.261	α-pleuene	0.24
2	6.395	α-pinene	3.4
3	6.677	Bicyclic [2,2,1] heptane	0.08
4	7.161	sabinene	3.51
5	7.232	β-pinene	12.79
6	7.338	Bicyclic [10,1,0] tridecane	0.08
7	7.458	β-myrcene	3.5
8	7.646	octanal	0.26
9	7.715	α-phellandrene	0.3
10	7.831	3-carene	0.2
11	7.946	4-carene	0.39
12	8.097	o-cymene	1.46
13	8.254	D-limonene	47.19
14	8.486	(Z)-3,7-dimethyl-1,3,6-octadecane triene	0.14
15	8.715	γ-terpinene	11.49
16	8.846	n-caprylic alcohol	0.08
17	8.935	2-vinyl-2-methyl-5-(1-methylvinyl) tetrahydrofuran	0.04
18	9.216	α-terpinolene	1.16
19	9.365	linalool	0.38
20	9.428	nonanal	0.17
21	9.975	6-methyl-3-(1-methylethyl)-7-oxicycle [4.1.0]-2-heptanone	0.18
22	10.046	D-litene oxide	0.1
23	10.167	3-oxacyclic [4.3.0] hept-8-ene-2-ketone	0.05
24	10.254	(R)-3,7-dimethyl-6-octenol	0.04
25	10.717	4-terpenenol	0.05
26	10.917	α-terpilenol	0.28
27	11.02	3-methylene-1,5,5-trimethylcyclohexene	0.05
28	11.088	capraldehyde	0.18
29	11.351	2-cyclohexene-1-ol, 2-methyl-5-(1-methylvinyl)-, mesylate	0.03
30	11.464	benzothiazole	0.05
31	11.536	2-cyclohexene 1-alcohol	0.06
32	11.67	(Z)-3,7-dimethylocta-2,6-dienal	2.81
33	11.751	D-carvone	0.03
34	12.113	(E)-3,7-dimethylocta-2,6-dienal	4.01
35	12.787	(2E)-1-methoxy-3,7-dimethyl acetamide	0.49
36	13.116	8-chloro-1-octanol	1.32
37	13.449	2,6-octadiene-1-alcohol-3,7-dimethylacetyl	0.75
38	13.715	geranyl acetate	0.57
39	14.449	caryophyllene	0.24
40	14.575	bergapten	0.45
41	14.923	humulene	0.06
42	15.263	Cyclohexene, 3-(1.5 dimethyl-4-hexene)	0.04
43	15.426	1,4-methylhydroindene	0.14
44	15.523	β-bisabolene	0.67
45	16.531	3,5-diethyl-2-propyl pyridine	0.03
46	17.358	Cyclooctane siloxane	0.06
47	19.226	Octadecymethyl cyclononsiloxane	0.03
48	20.673	m-camphorene	0.23
49	20.73	*N*-butyl phthalate	0.03
50	21.039	P-camphorene	0.1
Total			99.99

## Data Availability

The original contributions presented in the study are included in the article, and further inquiries can be directed to the corresponding author.
